# Immunotherapy and tumor mutational burden in cancer patients with liver metastases: A meta and real word cohort analysis

**DOI:** 10.3389/fonc.2022.994276

**Published:** 2023-01-19

**Authors:** Rui-Yan Wu, Bi-Cheng Wang, Kun Wang, Fan Xia, Zhi-Yuan Zhang, Jue-Feng Wan, Zhen Zhang

**Affiliations:** ^1^ Department of Radiation Oncology, Fudan University Shanghai Cancer Center, Shanghai, China; ^2^ Department of Oncology, Shanghai Medical College, Fudan University, Shanghai, China; ^3^ Shanghai Clinical Research Center for Radiation Oncology, Fudan University Shanghai Cancer Center, Shanghai, China; ^4^ Shanghai Key Laboratory of Radiation Oncology, Fudan University Shanghai Cancer Center, Shanghai, China; ^5^ Cancer Center, Union Hospital, Tongji Medical College, Huazhong University of Science and Technology, Wuhan, China

**Keywords:** immune-checkpoint inhibitors, liver metastases, tumor mutational burden (TMB), immunotherapy, meta - analysis

## Abstract

**Background:**

The predictive effects of liver metastases for immune-checkpoint inhibitors (ICIs) and the relationship between tumor mutational burden (TMB) and liver metastases (LM) remain unclear.

**Methods:**

A systematic review and meta-analysis were conducted to explore the heterogeneity of ICIs efficacy between patients with or without LM. A pan-cancer cohort of 1,661 patients who received ICIs was downloaded and analyzed to assess the association between TMB and LM.

**Results:**

Of 21053 studies identified in our search, eight single-arm studies and 24 randomized controlled trials were included. Overall, 17957 patients with advanced or metastatic cancers (4805 patients (26.8%) with LM and 13151 patients (73.2%) without LM) were enrolled. The pooled objective response rate (ORR) was 8.5% (95% CI 4%–13%) in the LM group versus 21% (95% CI 16%–21%) in the non-LM group. The pooled hazard ratio (HR) for death was 0.85 (95% CI 0.80–0.90) in the LM group treated with ICIs compared with the standard of care. In patients without LM who were treated with ICIs, the pooled HR for death was 0.78 (95% CI 0.73–0.82) compared with the standard of care. The difference in efficacy between patients with or without LM treated with ICIs was significant (p=0.04). Pan-cancer analysis revealed that the TMB-high rate was 10.8% in liver metastatic lesions versus 21.4% in other metastatic lesions (p=0.004). In addition, TMB was also significantly associated with OS as a binary cutoff (p=0.05) and was an independent prognostic variable (HR=0.98, P=0.047) as a continuous variable in patients with LM.

**Conclusions:**

In patients with LM, the efficacy of immunotherapy was attenuated, but TMB-high could predict better survival outcomes.

## Introduction

Cancer frequently metastasizes to the liver, and metastasizing of the liver contributed 30%-70% of cancer specific mortality (1). System therapies (including chemotherapy and target therapy) combined with local anatomic resections, radiation and interventional ablation were the standard therapeutics strategies for liver metastases. Given that patients with LM do not respond well to these conventional therapies and some were unable to continue due to liver dysfunction, these therapies offered a modest extension of survival.

ICIs targeting cytotoxic T-lymphocyte protein 4 (CTLA-4) and programmed death receptor-1 (PD-1) pathways have induced durable treatment responses in a wide variety of cancers. LM diminishes immunotherapy efficacy in preclinical models (2) and patients (2–4). Numerous trials showed that ICIs-based system therapy failed to obtain significant improvement in overall survival (OS) compared with the standard care in non–small cell lung cancer (NSCLC), small cell lung cancer (SCLC), and urothelial cancer (UCC) patients with LM (5, 6) (7, 8) (9). On the contrary, a favorable HR of death was shown in the renal-cell carcinoma (10), gastric, gastro-oesophageal junction, and oesophageal adenocarcinoma (11) patients with LM. Two meta-analyses done in advanced or metastatic cancers found no statistically significant association of LM with the efficacy of ICIs (12, 13). However, these meta-analyses included a limited number of trials and patients. Since the clinical trials showed contradictory results and the meta-analysis was not sufficiently powered, the clinical benefit of ICIs in patients with LM remains further investigated.

Higher TMB is associated with a higher number of tumor neoantigens that trigger a T cell response and clinically correlates with ICI outcomes (14, 15). A positive association of TMB and ICIs treatment efficacy was observed in advanced melanoma (16, 17), NSCLC (18, 19), gastroesophageal adenocarcinoma (20), and urothelial cancer (21). Samstein et al. reported that high TMB predicted superior survival across diverse types of human cancers (22), outside of these specific clinical trial populations. Whether high TMB predicts overall survival in patients with LM for ICIs treatment remains further investigated.

Here, we did a pan-cancer meta-analysis to assess the efficacy of ICIs both in patients with LM and patients without LM. We also analyzed a real-world cohort to explore the association between TMB and metastases sites and the predictive value of TMB on the efficacy of ICIs in the presence or absence of LM.

## Methods

### Search strategy and study selection

This analysis was conducted following the Preferred Reporting Items for Systematic Reviews and Meta-analyses (PRISMA) guideline (23).

We systemically searched PubMed, Embase, and Cochrane Library for published trials from January 01, 2011 to June 30, 2022, using the terms (“ipilimumab” OR “tremelimumab” OR “nivolumab” OR “pembrolizumab” OR “cemiplimab” OR “atezolizumab” OR “durvalumab” OR “avelumab” OR “tislelizumab” OR “sintilimab” OR “camrelizumab” OR “toripalimab”) AND (“trial” OR “clinical trial”) AND (“cancer” OR “carcinoma” OR “tumor” OR “tumour” OR “neoplasm”). Two authors (Ruiyan Wu and Kun Wang) independently conducted the searching process. The references of the included studies were manually searched for further eligible studies. Conference abstracts were excluded.

To be included, single arm studies had to assessed ORR for a single ICIs according to LM status; randomized trials had to meet the following criteria: (1) participants in the intervention group treated with a single ICIs or ICIs combinations or ICIs combined with standard care, and participants in the control group received standard care without ICIs; (2) have data available for the hazard ratio (HR) for death according to LM status. For studies with multiple reported data, we analyzed the most recent report and excluded the duplicates. All eligible studies were published in English. Any discrepancies were discussed and resolved by consensus.

### Data analysis

Basic characteristics concerning the first author, publication year, study design, study phase, primary tumor, line of therapy, study drugs, number of patients, age, sex, median follow-up time, HR for death in the overall population, ORR and HR according to patients LM status were collected. The primary endpoint was the difference of ORR and HR for death reported according to LM status. We derived the ORR and their 95% CIs from single-arm trials, HRs for death and their 95% CIs (separately in intervention group and control group) from randomized trials for patients with or without LM. Statistical heterogeneity was estimated using the Q tests and quantified the heterogeneity of the results using I2 statistic percentages. The pooled ORR and HR for death according to LM status were calculated using a fixed-effects model (Mantel-Haenszel method) if the heterogeneity test showed no statistical significance (I2 ≤ 50% or p ≥ 0.10). Otherwise, a random-effects model was adopted. The heterogeneity between two estimates HR was assessed by interaction test (24).

To ascertain the risk of bias, the Risk of bias (RoB2) tool was used to evaluate the quality of randomized studies. A controlled trial could be assessed as “Low risk of bias”, “Some concern” and “High risk of bias”.

### Real-world cohort identification and analysis

A published cohort with clinical and genomic data of 1,661 advanced cancer patients treated with ICIs therapy from the cBioPortal online database (https://www.cbioportal.org) was downloaded (22). 930 patients were included in the analysis with genomic profiling conducted in metastasis tissue and 731 patients with primary tissue profiling were excluded. TMB was calculated by normalizing the total number of somatic non-synonymous mutations to the total number of megabases sequenced. We compared the TMB between liver metastasis tissues and other metastases tissues using wilcox.test (continuous variable) and chi-square test (binary cutoff). Kaplan–Meier (KM) survival analysis was used to assess the survival differences. Univariate and multivariate Cox regression analyses of clinicopathological variables were performed to select candidate predictors of survival. The statistically significant variables (P < 0.05) in univariate Cox regression analyses were incorporated into the multivariate Cox for predicting OS.

All reported p values are 2-sided, and p<0.05 was considered statistical significance. We did all analyses using R (version 4.1.0).

## Results

**Figure 1:**
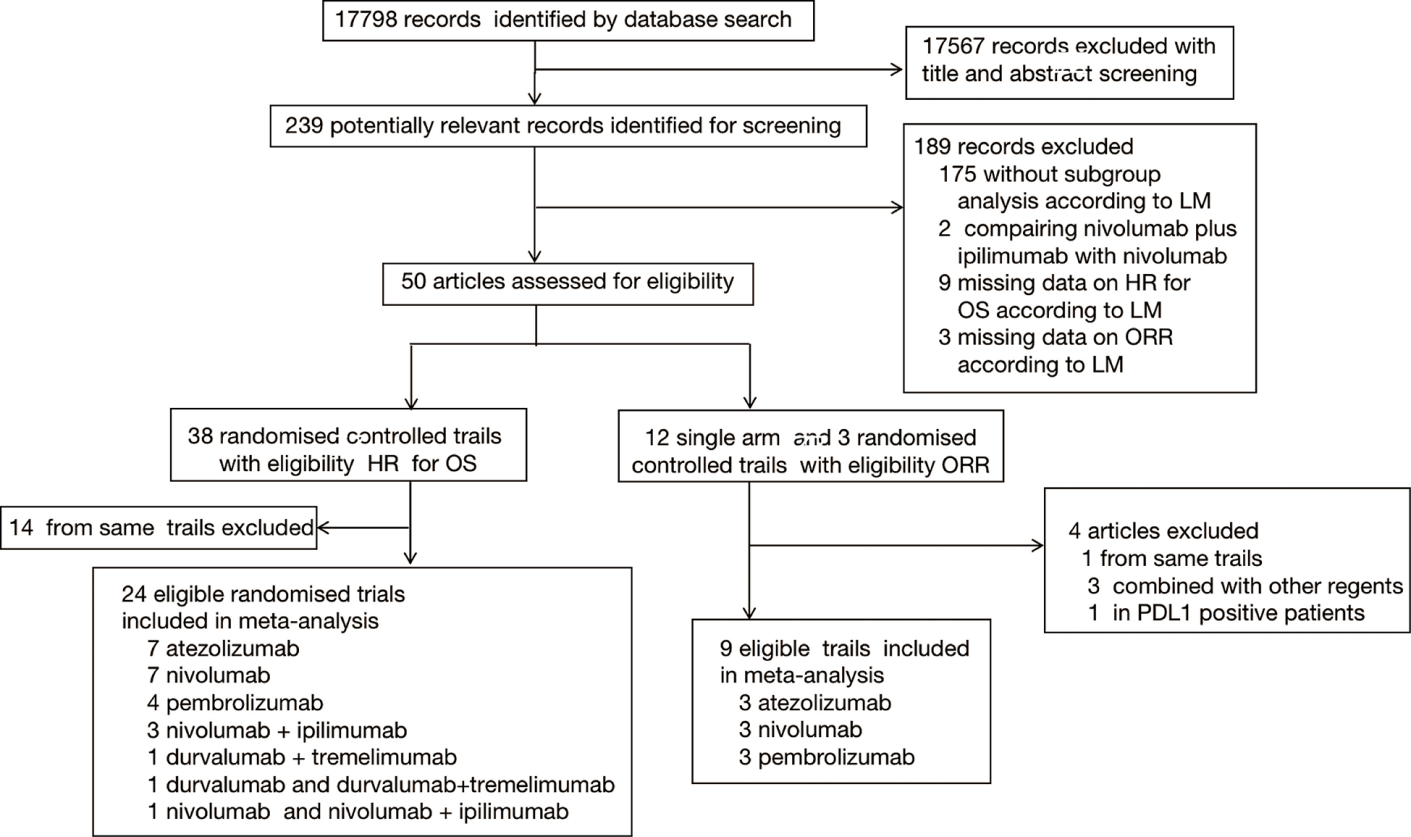
Study selection. Flowchart showing article identification, inclusion, and exclusion criteria.

A total of 21053 records (PubMed:6290; Embase:8792; Cochrane Library:5971) were identified. 17798 records were screened after duplicated (3255 records) removed, of which 239 were reviewed in full text. In total, 32 studies [8 single-arm studies (21, 25–31) and 24 RCTs (5–11, 32–48)] involving advanced and metastatic cancers patients were included for analysis (**Figure 1**). There were 18 trials with first-line therapy, 13 trials with second or additional lines of therapy, and 1 trial with maintenance therapy. The cancer types including in our analysis were non-small cell lung cancer (NSCLC, n=6), small cell lung cancer (SCLC, n=5), urothelial cancer (UC, n=4), oesophageal squamous cell carcinoma or gastric or gastro-oesophageal junction cancer (EC/GEJ/G, n=4), renal cell carcinoma (RCC, n=3), triple-negative breast cancer (TNBC, n=1) and prostate cancer (PC, n=1). Basic characteristics of the trials included in the systematic review and pooled analysis were summarized in **Supplementary Table 1** and **Supplementary Table 2**.

23 trials reported data on HR for death, and eight trials reported ORR according to LM status, one trial reported both HR for death and ORR, and the reported data was extracted for pooled HR for death and ORR analysis. 2 single-arm studies included cisplatin-ineligible patients. One trial enrolled IMDC intermediate- and poor-risk only UCC patients. We didn’t include studies reporting data on HR for death or ORR stratified by PD-L1 expression.

First, we explored whether LM could affect the response of ICIs. Of the total of 9 trials enrolling 2702 patients reported data on ORR, 740 patients with LM (27.4%) and 1962 patients without LM (72.6%). ICIs monotherapy had a significantly lower ORR patients with LM (pooled ORR, 0.08; 95% CI, 0.04-0.13) compared to patients without LM (ORR, 0.22; 95% CI, 0.17-0.27). There was substantial inter-study heterogeneity among single-arm studies estimates in both patients with LM (Q = 44.44, p<0 .001, I2 = 82.0%) patients without LM (Q = 64.15, p<0 .001, I2 = 87.5%) (**Supplementary Figure 1**).

**Figure 2:**
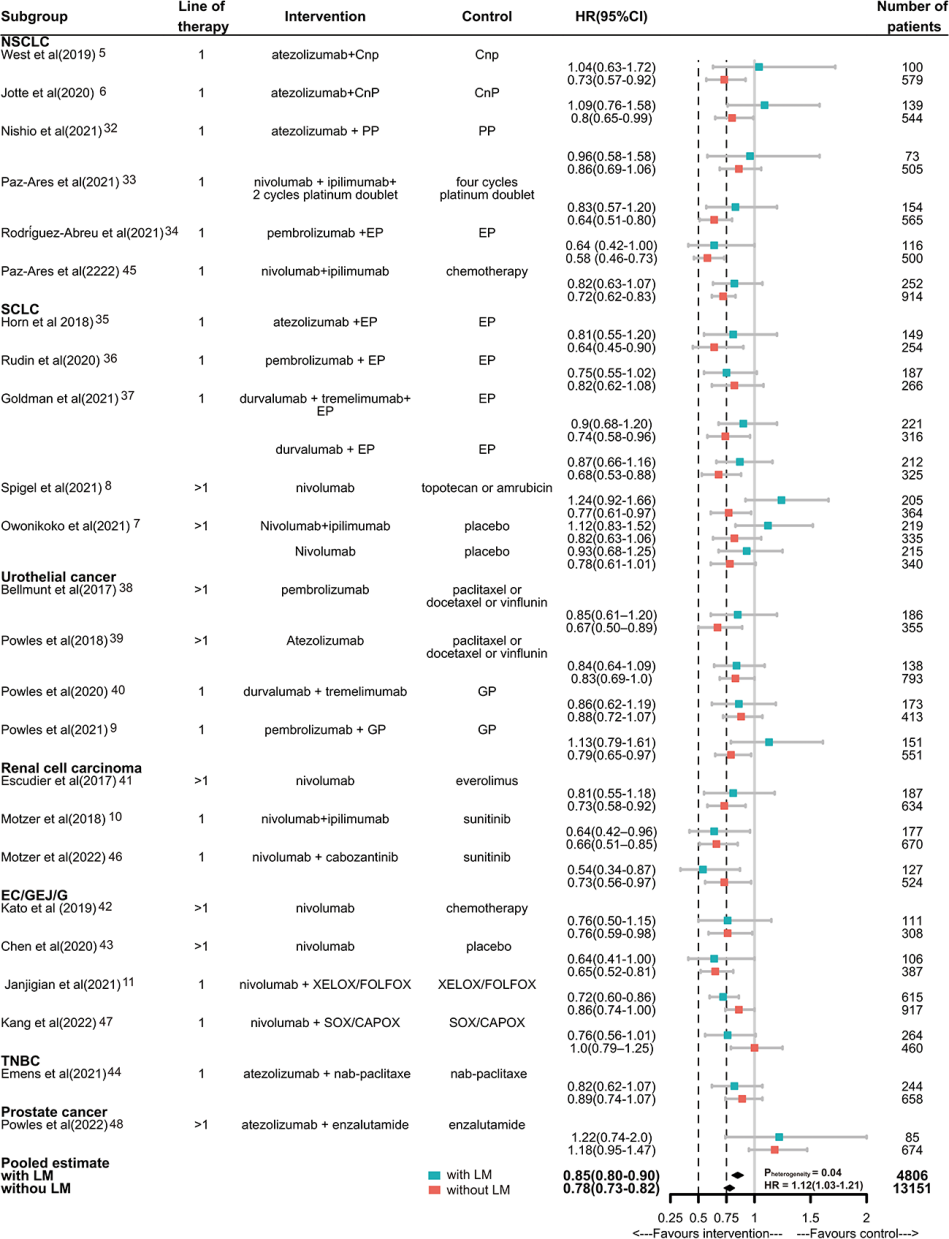
Hazard ratios of death for patients assigned to intervention treatment, compared with those assigned to control treatment, by LM status. Squares represent study-specific HRs. Horizontal lines indicate the 95% CIs. Diamonds indicate the meta-analytic pooled HRs, calculated separately in the presence or absence of LM, corresponding to 95% CIs. The p value for heterogeneity is from the meta-analysis of the interaction HRs and represents heterogeneity by LM status. Cnp100= carboplatin plus nab-paclitaxel. PP, pemetrexed plus cisplatin or carboplatin; EP/EC, etoposide plus cisplatin or carboplatin; GP, gemcitabine plus cisplatin or carboplatin; CAPOX/FOLFOX, capecitabine plus oxaliplatin or leucovorin, fluorouracil, plus oxaliplatin. HR, hazard ratio.

Then, we compared the survival benefit of ICIs versus standard care in the presence or absence of LM. Of the total of 24 trials enrolling 17957 patients reported data on HR for death, 4805 with LM (26.8%) and 13151 without LM (73.2%). For patients without LM, immunotherapy achieved a prolonged OS compared with control therapy (pooled HR, 0.78; 95% CI, 0.73-0.82). An OS advantage of immunotherapy was also obtained for patients with LM but was smaller (HR, 0.85; 95% CI, 0.80-0.90). There was significant inter-study heterogeneity among single study estimates in patients without LM (Q=43.08, p=0.01, I2 = 43%), but not in patients with LM (Q=30.62, p=0.20, I2 = 18%). There was a significant difference in the efficacy of ICIs between different LM status, when compared with controls (pheterogeneity=0.04, **Figure 2**). The pooled interaction HR (the pooled estimate of the ratios of the HRs in patients with LM and patients without LM reported in each trial) was 1.12 (95% CI 1.03-1.21). The RoB of 23 RCTs was generally low to moderate (**Supplementary Figure 2**).

**Figure 3:**
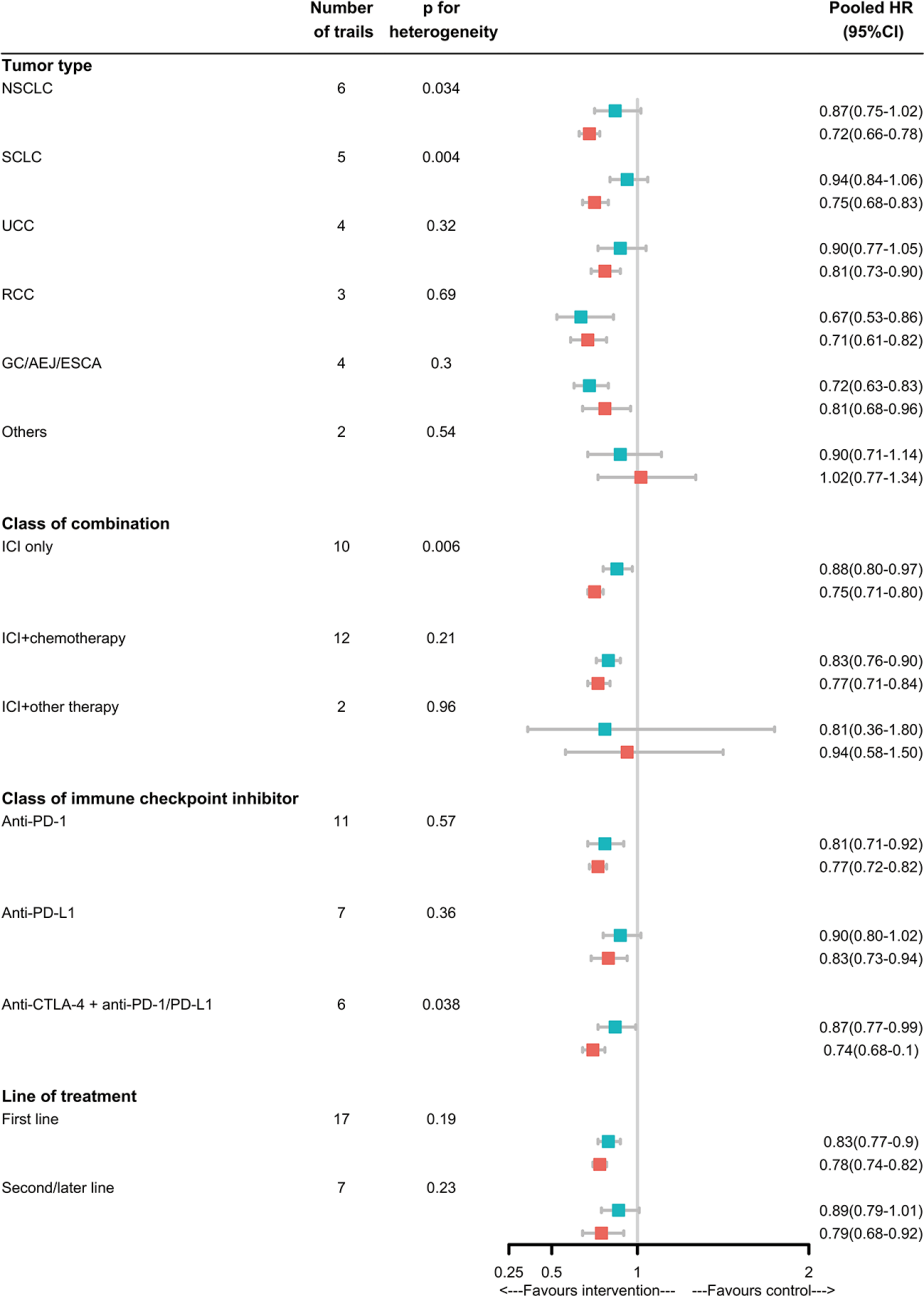
Analyses of LM-specific pooled hazard ratios, by subgroup. Squares represent subgroup-specific pooled hazard ratios (HRs). Horizontal lines indicate 95% CIs. The p-value for heterogeneity is from an interaction test that compares the estimated HRs across different LM statuses and represents heterogeneity within each subgroup.

In subgroup analyses, for NSCLC, SCLC, ICIs only without combination with chemotherapy or target therapy, anti-PD-1/PD-L1 plus anti-CTLA-4, the magnitude of efficacy of ICIs was greater for patients without LM than patients with LM, and the heterogeneity test for this LM-related interaction was significant. For UC, RCC, EC/GEJ/G, ICIs combination with chemotherapy therapy, anti-PD-1or anti-PD-L1, first or subsequent line of treatment, the efficacy of ICIs was not significant different. Notably, in TNBC and PC, the ICIs was not effective in reducing risk of death for patients with or without LM. But due to the limited trails available, we coudn’t draw specific conclusions(**Figure 3**).

**Figure 4:**
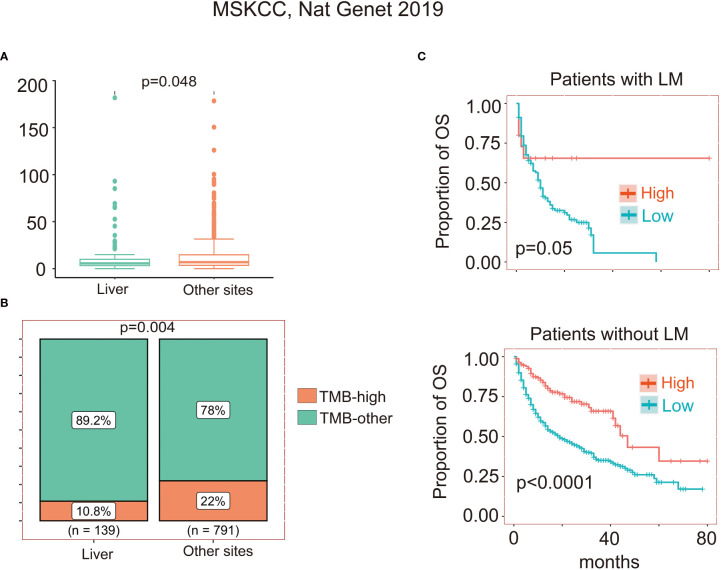
Association between TMB and metastases. **(A)**Analyses of TMB score between different metastases sites, the p value is from wilcox test. **(B)**Analyses of TMB-high and TMB-other distribution between different metastases sites, TMB-high was defined as the top 20% of patients and the bottom 80% patients were defined as TMB-other. The p value is from Chi-square test. **(C)** Kaplan-Meier graph of overall survival of 914 patients with overall survival longer than one month. Two-sided log-rank p value indicated the Top20% groups compared to the bottom80% group for patients with or without LM.

Finally, we evaluated the prognostic impact of TMB in different metastases tissues since TMB is widely used to predict clinical outcomes of ICIs. We identified a cohort of 1,661 cancer patients with 11 cancer types. We included 930 patients with TMB score estimated in metastasis tissues (liver metastasis: 139, other site metastasis: 791). No significant differences between the clinicopathological characteristics (age, sex, and drug type of ICIs), except cancer type and TMB, were observed in the presence or absence of LM (**Supplementary Table 3**). TMB score was higher in patients with LM (p=0.048) (**Figure 4A**). We performed a stratified analysis by binarizing TMB level as TMB-high (top 20%) and TMB-other (bottom 80%) and found that TMB-high rate was half smaller in patients with LM patients without LM (p=0.004) (**Figure 4B**). TMB-high corresponded to a favor OS in patients with LM (p=0.05) and patients without LM (p<0.001) by Kaplan-Meier (KM) survival analysis (**Figure 4C**). Then, we further investigated whether TMB was an independent prognostic variable in patients with LM. Univariate and multivariate Cox regression analyses of clinicopathological variables were performed to select candidate predictors of survival. The statistically significant variables (P < 0.05) in univariate Cox regression analyses were incorporated into the multivariate Cox regression to test each factor’s independence for prediction OS. TMB was significantly associated with OS as a continuous variable (HR=0.98; 95% CI, 0.97-1.00, p=0.047), but the binary cutoff (top 20%, HR=0.42; 95% CI, 0.17-1.05, p=0.064) was marginal (**Supplementary Table 4**). Then, after adjusting for cancer type and drug type of ICIs by multivariable analysis, TMB as a continuous variable was still independency variable for prediction OS in patients with LM (adjusted HR=0.98; 95% CI, 0.96-1.00, p=0.049) (**Supplementary Table 5**).

## Discussion

This study showed that ICI-based systemic therapy could improve overall survival for patients with or without LM. However, patients with LM had a smaller treatment benefit from these drugs versus control treatments than patients without LM. To our knowledge, this was the first study to clearly show significant heterogeneity in the efficacy of immune checkpoint inhibitors according to the patient’s liver metastases status. The benefit difference of our findings was strengthened by single-arm studies of ICIs monotherapy. The pooled ORR was smaller than half the size for patients with LM than for patients without LM. This result was clinical reverence to the previous report that patients with LM had significantly shorter overall survival (OS) than those without LM (10 *vs*. 20 months; P < 0.0001) (4).

The liver is naturally programmed as an immune privilege organ with hypo-reactivity to food-derived antigens and bacterial products through the portal vein (49–51). The classic hypothesis was that a variety of hepatic cell types can induce activated T-cell accumulation and apoptosis in the liver, including liver-resident antigen-presenting cells (APCs) (52), Kupffer cells (53), liver sinusoidal endothelial cells (54), hepatocytes (55), NKT cells (56) and stellate cells (57). Liver metastases further prompted hepatic immunotolerance by eliminating systemic tumor-specific CD8+ T cells through macrophages induced, Fas-FasL pathway dependent apoptosis (2). In fact, the presence of liver metastases was associated with fewer infiltrating CD8+ T cells at the invasive margin in distant tumors (3). Besides, Lee et al. reported that liver could lead to the systemic suppression of antitumor immunity and response to anti-PD-1 immunotherapy through activation of regulatory T cells (Tregs) and modulation of intratumoral CD11b+ monocytes (58). This general state of tolerance enabled the expansion of local metastases and had systemic consequences that manifested poor response to immunotherapy in patients with LM. Subgroup analysis from a total of 11 trials of NSCLC and SCLC showed that the efficacy of ICIs was evidently decreased and marginal for patients with LM. The fundamental inhibition mechanisms and magnitude of decreased efficacy, suggested that more assessment should be paid for patients with LM in the routine clinical practice of ICIs treatment, especially in NSCLC and SCLC.

Since specific adverse events and the significant economic cost of ICIs, efforts are an urgent need for identifying predictive biomarkers to select patients who would derive the maximum potential benefit from immunotherapies with liver metastases. We found that TMB, as a continuous variable, was an independent prognostic factor (HR=0.98, P=0.047) adjustment for cancer type and drug class of ICIs by multivariate Cox regression analyses for patients with LM. As a binary cutoff, TMB was also significantly associated with OS by Kaplan-Meier (KM) survival analysis (p=0.05) but the HR was marginal. We supposed that the contradiction result might due to limited patients number (n=15) in TMB-high group.

We also found that both TMB score and TMB-high rate was smaller in patients with LM, which might partly contribute to the relatively poor response. A previous pan-cancer analysis based on the same cohort of 1,661 cancer patients showed that TMB level was comparable between patients with and without LM (22). In our analysis, we included 930 patients with TMB estimated in metastasis tissue, the patients with TMB estimated in primary tissue were excluded for analysis. Besides, we compared the high TMB (defined as the highest 20%) rate instead of TMB level since the former is widely used in clinical practice. There were studies reported that the TMB status differed between the liver metastatic lesions, primary lesions and lung metastatic lesions. Hoshion et al. found that TMB was found to be high (10 or more per 1 Mb) in 8 out of 24 patients with primary lesions and in 5 of 24 patients with liver metastatic lesions (59). Wang et al. found the lung metastases from colorectal cancer (CRC) demonstrated notably higher TMB levels than those of liver metastases from CRC (6.022 *vs*. 2.02 SNVs/Mb, P=0.044) and a TMB >10 SNVs/Mb was observed more frequently in samples from the lung metastases than liver metastases cohort (P=0.004) (60).

Data from clinical trials (IMpower150 (61)) indicated that chemotherapy could restore systemic efficacy of ICIs for patients with LM. Our result showed that for ICIs combined with chemotherapy, comparable benefit (pheterogeneity=0.56) was yielded regardless of LM status, while for ICIs only without combination, the benefit was marginal and evidently decreased (Pheterogeneity=0.006) in patients with LM. These data were in line with the hypothesis that tumor cell destruction by chemotherapy may provide a broad neoantigen to facilitate immune recognition and anti-tumor immune response (62). Radiotherapy could reshape the liver immune microenvironment *via* increasing hepatic T cell infiltration, diminishing liver myeloid cell number and diminishing the ratio of CD11b+F4/80+ myeloid cells to CD8+ T cells, thus abolished immunotherapy resistance induced by liver metastasis in MC38 mouse modle (2). With appropriate biomarker and manipulation of the liver metastases microenvironment, a positive response of ICIs for patients with LM could be elicit.

Subgroup analysis showed that, anti-PD-1/PD-L1 combined with CTLA-4 can significantly improve overall survival in both patients with or without LM, but the magnitude of this benefit is largely dependent on liver metastasis status. For patients with LM, the efficacy of ICIs was valid for patients in anti-PD-1 and first-line settings but was marginal in anti-PD-L1 and second-line settings. The biological difference between anti-PD-1 and anti-PD-L1 attributed to disrupting different aspects of the same ligand-receptor interaction: blockade of additional ligand-receptor interactions such as PD-L2-PD-1 with anti-PD-1, but not with anti-PD-L1 (63). Human adult liver expressed PD-L2 but not PD-L1 (63), and these might relate to the benefit difference between anti-PD-1 and anti-PD-L1 in patients with LM. In addition, chemotherapy resistance increased the level of terminally differentiated and immunosuppressive cells (i.e., SPP1+ and MRC1+ CCL18+ macrophages) while decreased the cytotoxic immune cells (i.e., FGFBP2+ GZMB+ CD8+ T cells) in liver metastases patients (64), providing evidence to support first-line use of ICIs for patients with LM. For these reasons, anti-PD1 or combination with an-CTLA-4, the first-line setting was more favorable for patients with LM.

This study had several limitations. Firstly, our meta-analysis relied on published results rather than on individual patients’ data and we could not exclude the variables that might influence our results. Apart from TMB, factors associated with ICIs efficacy, such as age, gender, the expression of PD-L1 or the EGFR or ALK mutational status, were not so far known to be distributed differentially associated with patients’ liver metastases status. Secondly, a relatively small number of eligible trials for RCC, EC/GEJ/G, TNBC and PC were included in the analysis and we cannot make a solid conclusion on the true predictive or prognostic significance of LM in the tumor type mentioned above. Thirdly, no eligible trial for melanoma patients was included in our analysis due to no RCT reported HR for death according to liver metastases status.

Two main conclusions can be drawn from this study. First, LM was an important prognostic variable and should be considered in the assessment of risk versus benefit for ICI-based systemic therapy. ICI-based systemic therapy could significantly improve overall survival in both patients with or without LM but the magnitude of this benefit is largely LM status dependent. Future research should focus on improving outcomes for patients with LM. Second, high TMB was distributed differentially according to LM status and was also a favor biomarker for ICI-based systemic therapy regardless of LM status. However, due to the relatively small number of eligible patients into the final analysis, the prognostic value of high TMB in the presence of LM remained further investigated.

## Data availability statement

The original contributions presented in the study are included in the article/**Supplementary Material**. Further inquiries can be directed to the corresponding authors.

## Author contributions

Study design: ZZ, J-FW and R-YW. Data extraction: R-YW, B-CW, KW, FX, Z-YZ. Data analysis: R-YW, B-CW, KW, FX, Z-YZ. Manuscript writing: R-YW, B-CW, KW. Manuscript edition: ZZ, J-FW. All authors contributed to the article and approved the submitted version.
