# Supplementary material for: Immunotherapy and tumor mutational burden in cancer patients with liver metastases: A meta and real word cohort analysis
Source: Front Oncol. 2023 Jan 19;12:994276. doi: 10.3389/fonc.2022.994276 (PMC9893030; doi:10.3389/fonc.2022.994276)
Supplement: Supplementary file 5 [file Table_3.docx]

**Supplementary Table 3** Demographic and clinical characteristics of patients with LM or without LM.

| **Characteristics** | **Liver metastase(139)** | **Other site metastase(791)（791）（（（791** | p |
| --- | --- | --- | --- |
| **Age** |  |  | .373 |
| Median（**IQR)** | 629(51-71) | 63(54-71) |  |
| **Sex** |  |  | .814 |
| Female | 56 (40.3) | 307 (38.8) |  |
| Male | 83 (59.7) | 484 (61.2) |  |
| **Cancer Type (%)** |  |  | .000 |
| Bladder Cancer | 13 (9.4) | 79 (10.0) |  |
| Breast Cancer | 6 (4.3) | 27 (3.4) |  |
| Cancer of Unknown Primary | 13 (9.4) | 69 (8.7) |  |
| Colorectal Cancer | 26 (18.7) | 34 (4.3) |  |
| Esophagogastric Cancer | 9 (6.5) | 38 (4.8) |  |
| Glioma | 0 (0.0) | 2 (0.3) |  |
| Head and Neck Cancer | 8 (5.8) | 89 (11.3) |  |
| Melanoma | 31 (22.3) | 235 (29.7) |  |
| Non-Small Cell Lung Cancer | 31 (22.3) | 148 (18.7) |  |
| Renal Cell Carcinoma | 2 (1.4) | 69 (8.7) |  |
| Skin Cancer, Non-Melanoma | 0 (0.0) | 1 (0.1) |  |
| **Drug Type (%)** |  |  | 0.409 |
| Combo | 31 (22.3) | 141 (17.8) |  |
| CTLA4 | 10 (7.2) | 70 (8.8) |  |
| PD-1/PDL-1 | 98 (70.5) | 580 (73.3) |  |
| **Gene Panel** |  |  | 0.782 |
| IMPACT341 | 15 (10.8) | 102 (12.9) |  |
| IMPACT410 | 88 (63.3) | 485 (61.3) |  |
| IMPACT468 | 36 (25.9) | 204 (25.8) |  |
| **TMB score (%)** |  |  | 0.005 |
| High | 15 (10.8) | 171 (21.6) |  |
| Non-high | 124 (89.2) | 620 (78.4) |  |

| **Supplementary Table 4** Univariate Cox regression for clinical prognostic values in patients with LM. | | | | | |
| --- | --- | --- | --- | --- | --- |
| Characteristic | number | HR | 95% CI | | *P*-value |
| TMB Continuous | 139 | 0.98 | 0.97 | 1.00 | 0.047 |
| TMB Binary(low) |  |  |  |  |  |
| Low | 124 | - |  |  |  |
| High | 15 | 0.42 | 0.17 | 1.05 | 0.064 |
| Age | 139 | 1.00 | 0.98 | 1.01 | 0.643 |
| Cancer type |  |  |  |  |  |
| Bladder cancer | 13 | - |  |  |  |
| Breast Cancer | 6 | 0.34 | 0.09 | 1.23 | 0.1 |
| Colorectal Cancer | 26 | 0.55 | 0.25 | 1.20 | 0.132 |
| Esophagogastric Cancer | 9 | 0.60 | 0.19 | 1.91 | 0.391 |
| Head and Neck Cancer | 8 | 1.39 | 0.51 | 3.81 | 0.520 |
| Melanoma | 31 | 0.25 | 0.11 | 0.57 | 0.001 |
| Non-small Cell Lung Cancer | 31 | 0.66 | 0.32 | 1.36 | 0.260 |
| Renal Cell Carcinoma | 2 | 0.28 | 0.04 | 2.21 | 0.229 |
| Cancer of Unknown Primary | 13 | 1.23 | 0.51 | 2.98 | 0.644 |
| Drug type |  |  |  |  |  |
| Combine | 31 | - |  |  |  |
| CTLA-4 | 10 | 1.39 | 0.55 | 3.53 | 0.488 |
| PD-1/PD-L1 | 98 | 1.99 | 1.10 | 3.62 | 0.023 |
| Gene panel |  |  |  |  |  |
| IMPACT341 | 15 |  |  |  |  |
| IMPACT410 | 88 | 0.93 | 0.49 | 1.75 | 0.812 |
| IMPACT468 | 36 | 0.66 | 0.31 | 1.40 | 0.277 |
| Gender |  |  |  |  |  |
| Femal | 56 | - |  |  |  |
| Male | 83 | 1.462 | 0.686 | 3.112 | 0.325 |
| The control group for Cox regression in each clinical value was labeled by ‘-’ symbol. HR, Hazard radio, CI,confidence interval. | | | | | |

| **Supplementary Table 5** Clinical prognostic values in patients with LM by Multivariate Cox regression. | | | | |
| --- | --- | --- | --- | --- |
| Characteristic | HR | 95% CI | | *P*-value |
| TMB Continuous | 0.98 | 0.96 | 1.00 | 0.049 |
| Cancer type |  | | | |
| Bladder cancer | - |  |  |  |
| Breast Cancer | 0.20 | 0.05 | 0.85 | 0.030 |
| Colorectal Cancer | 0.51 | 0.23 | 1.13 | 0.097 |
| Esophagogastric Cancer | 0.61 | 0.19 | 1.94 | 0.403 |
| Head and Neck Cancer | 1.29 | 0.46 | 3.60 | 0.622 |
| Melanoma | 0.24 | 0.10 | 0.57 | 0.001 |
| Non-small Cell Lung Cancer | 0.62 | 0.30 | 1.31 | 0.212 |
| Renal Cell Carcinoma | 0.25 | 0.03 | 1.97 | 0.189 |
| Cancer of Unknown Primary | 1.04 | 0.42 | 2.57 | 0.928 |
| Drug type |  | | | |
| Combine | - |  |  |  |
| CTLA-4 | 2.37 | 0.87 | 6.46 | 0.092 |
| PD-1/PD-L1 | 1.10 | 0.54 | 2.23 | 0.786 |
